# Cyanobacteria vs green algae: which group has the edge?

**DOI:** 10.1093/jxb/erx226

**Published:** 2017-09-05

**Authors:** John Beardall, John A Raven

**Affiliations:** 1 School of Biological Sciences, Monash University, Clayton, Victoria, Australia; 2 Division of Plant Sciences, University of Dundee at the James Hutton Institute, Invergowrie, Dundee, UK

**Keywords:** Algal blooms, carbon dioxide, climate change, CO_2_-concentrating mechanism, competition model, cyanobacteria, green algae, lakes, *Microcystis*

## Abstract

This article comments on:

**Ji X, Verspagen JMH, Stomp M, Huisman J.** 2017. Competition between cyanobacteria and green algae at low versus elevated CO_2_: who will win, and why? Journal of Experimental Botany **68,** 3815–3828.

**The dogma surrounding carbon assimilation has it that, due to their highly effective CO**_**2**_ **-concentrating mechanisms, cyanobacteria will always out-perform, for example, green algae where inorganic carbon is in short supply. Working on the cyanobacterial genus *Microcystis*,** Ji *et al.* (2017 **) now suggest this might not always be true, with possible improved performance with rises in atmospheric (and hence dissolved) CO**_**2**_ **. Many cyanobacteria form extensive toxic blooms that present significant health risks and economic costs: how they will react in a future world with elevated CO**_**2**_ **and temperature is thus of intense interest for water management.**

Cyanobacteria and algae possess various inorganic carbon transporters (CO_2_-concentrating mechanisms, CCMs) that serve to increase the CO_2_ concentration at the active site of Rubisco (ribulose-1,5-bisphosphate carboxylase oxygenase). CCMs presumably evolved because the CO_2_-fixing enzyme has a relatively low catalytic rate and expresses a competitive oxygenase as well as the carboxylase activity, with the rates of the two activities depending on the O_2_:CO_2_ ratio at the active site of the enzyme, according to Eqn (1):

$$S_{rel}=(K_{0.5}\left(O_{2}\right)k_{cat}(CO_{2}))/(K_{0.5}\left(CO_{2}\right)k_{cat}(O_{2}))$$ (1)

where the selectivity factor *S*_rel_ defines the ratio of rates of carboxylase to oxygenase reactions, *k*_cat_ (CO_2_) = CO_2_-saturated specific rate of carboxylase activity of Rubisco (mol CO_2_ mol^–1^ active site s^–1^), $K_{0.5}$ (CO_2_) = concentration of CO_2_ at which the CO_2_ fixation rate is half of *k*_cat_ (CO_2_), *k*_cat_ (O_2_) = O_2_-saturated specific rate of oxygenase activity of Rubisco (mol O_2_ mol^–1^ active site s^–1^) and $K_{0.5}$ (O_2_) = concentration of O_2_ at which the O_2_ fixation rate is half of *k*_cat_ (O_2_).

A number of different forms of Rubisco, with a range of kinetic properties, occur in autotrophic organisms (Badger *et al.*, 1998; Raven and Beardall, 2003; Beardall and Raven, 2016). In short, freshwater cyanobacteria tend to have Rubiscos with high *K*_0.5_ (CO_2_) and *k*_cat_, and low *S*_rel_, values whereas green algae have Form 1B Rubiscos with higher affinity [lower *K*_0.5_ (CO_2_)] and *S*_rel_ but lower *k*_cat_ (Raven and Beardall, 2003). Differences in the kinetic properties of Rubisco among species mean that the different forms of Rubisco will perform differently at a given set of CO_2_ and O_2_ concentrations at the active site. Thus, at present-day dissolved CO_2_ levels, organisms with low affinity for CO_2_ [high *K*_0.5_ (CO_2_)] will have Rubiscos operating well below maximum capacity if internal CO_2_ is in equilibrium with (or lower than) external CO_2_; indeed, species such as dinoflagellates, with their low *S*_rel_ Form II Rubisco would probably be incapable of performing net C assimilation with diffusive CO_2_ entry at air equilibrium (Beardall and Raven, 2016). Although some algal species are capable of functioning well with diffusive CO_2_ entry, these tend to be restricted to environments where CO_2_ levels are high – as is the case for the freshwater red algae belonging to the Batrachospermales (Raven *et al.*, 1982), the Chrysophytes *sensu lato* (Maberly *et al.*, 2009), and the coccoid symbiotic green alga *Coccomyxa* using CO_2_ from soil or basiphyte respiration (Raven and Colmer, 2016) – or where low light levels constrain photosynthesis so CO_2_ diffusion is sufficient to satisfy demand (Kübler and Raven, 1994, 1995). In all other cases examined, net CO_2_ assimilation by cyanobacteria and algae requires the operation of a CCM, which increases the CO_2_ supply to the active site of Rubisco.

## Not all CCMs are equal

In general terms, and as a consequence of the lower affinity of their Rubiscos for CO_2_, cyanobacteria tend to show higher expression of CCM activity (based on internal:external CO_2_ concentration ratios) compared to green algae and this, together with observations of preferences of cyanobacteria for high pH environments where the proportion of CO_2_ relative to bicarbonate is low, is taken as suggesting a greater competitive ability by cyanobacteria when CO_2_ levels are low. As pointed out by Ji *et al.* (2017), there is some evidence for this from ecological observations (Shapiro, 1990, 1997) as well as previous competition experiments with freshwater phytoplankton communities (Low-Décarie *et al.*, 2011, 2015), though Caraco and Miller (1998) caution that high pH could be as important a driver to the competitive success of cyanobacteria as CO_2_.

Such generalizations, however, tend to ignore the variability among CCMs and specifically the range of transporters used for inorganic carbon acquisition. Thus cyanobacteria can express up to five different transporters of inorganic carbon with differing capacity, substrates and affinity. These are summarized in Box 1.

**Box 1. Characteristics of cyanobacterial DIC transporters**

Cyanobacterial inorganic carbon transporters differ in affinity and flux rate, and include HCO_3_^–^ transporters at the plasmalemma and CO_2_ transporters at the thylakoid membrane. Some cyanobacteria can express multiple transporters at the same time or can change expression patterns depending on, for example, external CO_2_ levels (Price, 2011; Sandrini *et al.*, 2015). Expression of different transporters among species and strains will thus confer different physiology and competitive capacity.

**Table:** 

Transporter	Substrate	Affinity	Flux	Notes
BCT1	HCO_3_^–^	High	Low	ABC-type transporter found exclusively in freshwater β-cyanobacteria; low-CO_2_ inducible
SbtA	HCO_3_^–^	High	Low	Sodium-dependent transporter
BicA	HCO_3_^–^	Low	High	Sodium-dependent transporter
_NDH-13_	CO_2_	High	Low	Energized conversion of CO_2_ to HCO_3_^–^
_NDH-14_	CO_2_	Low	High	Energized conversion of CO_2_ to HCO_3_^–^

What is also apparent in a number of systems is that in addition to physiological plasticity within a given strain, there is also genetic heterogeneity within cyanobacterial strains of the same species. In the case of *Microcystis* responses to light, for instance, Kardinaal *et al.* (2007) suggested that the shift from toxic to non-toxic strains during blooms can be explained by a difference in their ability to compete for light. For inorganic carbon use, Sandrini *et al.* (2014, 2015) and Visser *et al.* (2016) have shown that, for a number of cyanobacterial genera and species, strains exist that express genes for different combinations of the five transport systems shown in Box 1. Given that these different transporters confer different properties related to inorganic carbon uptake under different CO_2_/HCO_3_^–^ concentrations, different strains might be expected to respond differently to changes in CO_2_ levels. This expectation was recently confirmed. Sandrini *et al.* (2016) showed, in selection experiments and a lake study, that the strain composition of *Microcystis* adapts to rising CO_2_ levels. Natural selection favours *bicA* + *SbtA* strains in dense blooms in which CO_2_ is depleted, while *bicA* strains benefit from high CO_2_ concentrations. The CCMs of green algae have not been as extensively characterized as those of cyanobacteria, but, in general, accumulation factors (CO_2_ in:CO_2_ out) for chlorophytes are much lower (Raven and Beardall, 2003). This does not necessarily make them poor performers at low CO_2_ as the *K*_0.5_ (CO_2_) for their Rubiscos is lower than that of cyanobacteria.

This is where the work reported by Ji *et al.* (2017) comes in. They took a strain of the toxic cyanobacterium *Microcystis* which expresses *bicA*, a low affinity, high flux transporter (Box 1), and three green algal species, *Scenedesmus obliquus*, *Monoraphidium griffithii* and *Chlorella vulgaris,* and grew them in monoculture and then in various combinations in competition at low (100 ppm) and high (2000 ppm) CO_2_ levels. The monoculture experiments were used to provide parameters for a resource competition model designed to predict how the species would react to the dynamic changes occurring during growth in the mixed populations.

Ji *et al.* (2017) showed that at low CO_2_, all species were DIC limited, but the performance in terms of the ability to cope with low CO_2_ and to compete for HCO_3_^–^ ions was *Scenedesmus*>*Chlorella*>*Microcystis*>*Monoraphidium*. At high CO_2_, however, population density increased to the extent that cultures became light limited and the competitive capacity was then *Microcystis≈Scenedesmus*>*Chlorella*> *Monoraphidium.* When pairs of species were placed in competition at low or high CO_2_, the predictions based on the single species cultures were borne out. So at low CO_2_, the bicA transport system of the *Microcystis* strain did not confer a competitive advantage over the green algae, and at high CO_2_ the superior ability of *Microcystis* to cope with the intense shading in dense culture allowed it to outcompete the other species.

## Perspectives

It would be interesting to see how the competition between green algae and cyanobacteria would work out with cyanobacterial species/strains expressing higher affinity transporters such as SbtA or BCT1. The work of Sandrini *et al.* (2016) and Ji *et al.* (2017) implies that as the DIC concentrations in the water column change, we are likely to see different strains of cyanobacteria, expressing different transport systems, appearing and disappearing, with strains such as the *Microcystis bicA* strain used by Ji *et al*. becoming more dominant as atmospheric CO_2_ levels continue to rise. Although past studies have implied that elevated CO_2_ is likely to stimulate growth of green algae and other species such as diatoms or Chrysophytes with a lesser (or no) CCM activity (as reflected in internal:external CO_2_ concentrations) compared to cyanobacteria, it may well be that instead, all other things being equal, we will see a dominance of different cyanobacterial strains filling a succession of niches with varying conditions of alkalinity, pH and CO_2_/HCO_3_^–^ concentrations. Certainly such niche exploitation by different strains of cyanobacteria is used, for instance, in *Cylindrospermopsis raciborskii* (Burford *et al.*, 2016) and *Microcystis* (Kardinaal *et al.*, 2007) in relation to light availability.

A further complication to note is that CCM expression is not constant (except for constitutive expression of SbtA in the marine α-cyanobacteria such as *Prochlorococcus*; Badger and Price, 2003) and is likely to be modulated by a range of factors including light availability and nutrient levels as well as CO_2_ (Beardall and Giordano, 2002; Raven *et al.*, 2011; Raven and Beardall, 2014; Sandrini *et al.*, 2015; Maberly and Gontero, 2017). Thus the competition outcomes in the real world are likely to be much more complicated than the relatively simple systems Ji *et al*. used. Nonetheless, this work is a significant and useful advance in understanding and modelling possible consequences of competition between phytoplankton in a changing environment, and can be complemented by experimental evolution studies to take into account genetic adaptation (Raven and Beardall, 2016; Sandrini *et al.*, 2016).
